# Report of Dr. N. S. Davis, M.D.

**Published:** 1854-06

**Authors:** 


*Report of the Chairman of the Committee on Epidemics and the Sanitary Condition of Chicago, read to the regular meeting of Cook County Medical Society, June 13th*, 1854, by N. S. Davis, M. D., etc. etc.

The Committee, of which I am a member, was required to report every month, first in relation to any epidemic diseases that might occur under their own observation, and second in relation to the general sanitary condition of the city. In fulfilling my part of the duty imposed on the Committee, it is proper to remark that I have had no opportunity to consult my professional brethren in relation to facts that have occurred in their practice during the last month, and consequently my report this evening, must include only what has come directly under my own observation.

My present report will embrace a period of two months, reaching from the middle of April to the present time, (June 13th, 1854.)

During the latter part of winter and all the spring, the Scarlatina and Rubeola have prevailed in the city, and yet not producing a sufficient number of cases at any one time to merit the name of epidemic.

During the last two months, I have met with several cases of Rubeola, only two of which presented any other than ordinary symptoms. These two were characterized by the usual *catarrhal* symptoms during the first three days with moderate fever. On the morning of the fourth day the first patient, a little girl about 3 years old, coughed more severely and with a more stridulous sound than usual, and in a few hours after, all the symptoms of severe croup were developed, causing very great dyspnœa, restlessness and loud ringing, or stridulous cough. Only a few spots of eruption had made their appearance on the face, and the febrile action was not very high. Believing the symptoms of croup to be chiefly spasmodic, I directed the patient half a grain of the sub-sulphate of mercury (turpeth mineral) to be repeated every 15 minutes until vomiting was induced; after which an anodyne and diaphoretic powder, consisting of Pulv. Doveri 1 gr and Pulv. Antimonialis ½ gr. was to be given and repeated every hour, until the patient became relieved. The emetic afforded prompt relief, and before three of the last mentioned powders had been given, the cutaneous eruption made its appearance copiously over the surface of the body, and the patient had no return of the laryngeal difficulty.

The second case was a little boy about 5 years of age. The symptoms of croup were developed suddenly and very severely on the morning of the fourth day after the Catarrhal symptoms had commenced. The difficulty of breathing was very great, and the paroxysms of coughing very suffocating and stridulous; but the febrile symptoms and activity of the pulse were not presented in that degree corresponding with the severity of the croup. The eruption had appeared on the face and neck, sufficient to show its nature. The suddenness of the attack, the moderate amount of febrile action, and its occurrence at a time when the nervous system was highly susceptible from the specific influence of Rubeola, led me to regard this case like the first as spasmodic in its nature. I gave the patient at once 1 gr. of Sub Sulphate of Mercury, which produced free emeses within twenty minutes. The paroxysms of vomiting resulting from this dose were repeated at short intervals for three quarters of an hour, and with great relief to the breathing of the patient. I then directed a mixture of compound honey of squills and senega ℥j., and tincture of belladona ʒj., of which 10 drops were to be given every one, two or three hours, according to the severity of the symptoms. During the following twenty-four hours the symptoms of croup all subsided, the eruption of measles became more full, and the patient recovered without any other untoward symptoms.

Only six cases of scarlatina have come under my observation during the last two months. Nearly all of them were of the Anginose variety, with much swelling of the cervical glands, and general prostration. Only one of the cases terminated fatally, which was a little boy two years of age. The tonsils, parotid and lymphatic glands became much swollen with the first appearance of the eruption; producing great difficulty of breathing, restlessness, rapidity of pulse and dry skin. Within three days the fauces and tonsils became ulcerated and assumed that ash-gray color indicative of approaching gangrene. This condition rapidly spread over the whole interior of the mouth and lips; the face became pale, the pulse very rapid and feeble, and the patient died about the fifth day.

Another case, a little girl about five years old, occurred in the same family at the same time. The disease presented the same form and tendencies as in the younger child, but was less severe. The ulcerations in the mouth and fauces were limited in extent, though unhealthy in appearance and very slow to heal The glands behind and below the angle of the jaw were much swollen and tender, and the skin presenting a whiteish or shining aspect ; the pulse 120 per minute, small, and quick. I gave the following, viz :

℞. Tinct. Belladona ʒj.

Spits. Nit. Dulc ℥j , mixed, fifteen drops every four hours, and a powder consisting of

℞ Pulv. Doveri 1 gr.

Sub. Murias Hydrag. 1 gr. mixed, each night. Locally her mouth was washed with a solution of chloride of zinc one grain to two ounces of water, and the swollen glands externally constantly covered with cloths wet in an infusion of aconite leaves with muriate of amonia. Under this treatment she passed through the active stage of the disease, the eruption and fever disappeared. the tonsils and glands on the right side of the neck returned nearly to their natural condition, but a large swelling directly behind the angle of the jaw remained on the left side, presenting a pale and shining surface and an obscure feel of fluctuation over the most prominent part. The face and lips were also pale, the pulse small, quick and irritable, with much general debility and entire loss of appetite. I opened the swelling and gave exit to a considerable quantity of unhealthy looking pus, and directed half a tablespoonful of cod-liver oil each night and each morning and ten drops of the following mixture every four hours, viz :

℞. Aromatic Sulph. Acid, ℥j.

Sulph Quinine, 10 grs.

Sulph. Morph, 5 grs. mixed, and a diet of beef tea, milk, etc. The abscess continued to discharge freely, and on the third day after it was opened, a large tough string of gangrenous cellular tissue was removed through the opening. The patient remained feeble and irritable with rapid emaciation. The cod-liver oil induced nausea and was sometimes rejected by vomiting. It was consequently discontinued. The other tonic mixture was continued, and to secure the introduction of more nourishment into the system I directed a tablespoonful of alcohol and a little sugar to be added to a tea-cupful of sweet milk, and a tablespoonful to be given every two hours. The effect was very marked and favorable. The pulse was diminished in frequency, the skin became more moist, and she slept quietly nearly all the following night. Clothes wet in an infusion of aconite leaves with muriate of ammonia were continued on the swelled glands, and the patient continued rapidly to improve until the health was fully restored. The other four cases were marked by no striking peculiarity.

The only remaining disease to which I would call your attention is the spasmodic or epidemic cholera. During the last two months I have seen within the limits of the city 33 well marked cases, of which 15 were fatal.

The first six cases occurred between the 20th and 25th of April. All of them were characterized by copious *rice water* discharges from the bowels, to which was added in three of them, vomiting, cramps in the lower extremities, corrugation of skin, huskiness of voice, and suppression of urine. They occurred in different sections of the city and without any possible connection with each other. All but one of these patients had resided in the city several months, and some of them several years. Only one died; viz: an irish woman passed the middle period of life. She had come from New Orleans to this city by way of the Mississippi river. She arrived here on Monday evening and was attacked with violent vomiting and purging of rice-water, cramps, etc., on the Wednesday evening following. On Thursday morning I was called, and found her in complete collapse with every characteristic feature of that stage of cholera. A very partial degree of re-action, however, came on during the day and she lingered in a dull typhoid state nearly three days. She was in a shanty in the south west division of the city, with three or four shanties, occupied by irish residents, close around it. But no other cases occurred in that immediate neighborhood.

From the 25th of April to the 15th of May, I saw no well marked cases. Two or three fatal cases, however, occurred during that period, in the practice of other physicians.

Between the 15th and 20th of May I met with *five* highly malignant cases. The first was a young man, native of Scotland, who arrived here about ten days before his death. He came through Canada direct to this city. He began to have a moderate watery diarrhœa within two or three days after he arrived. This continued without receiving any attention on his part until the afternoon of May 16th, when the symptoms of cholera commenced with great violence. I saw him about four hours after the severe symptoms commenced, and found him in complete collapse. He died about two o'clock the same night.

One hour after his death I was called to a girl aged 6 years, in the same house. She had been affected with diarrhœa for two or three days, and was seized with copious rice-water evacuations up and down, with cramps in the extremities, etc., etc., early in the evening. When I saw her, at three o'clock A. M., there was no pulse at the wrist; the skin was cold, corrugated, and purple; the breath and tongue cold; craving for cold drink; eyes deeply sunken and conjunctiva injected with blood; the discharges and cramps had nearly ceased; but still most of her drink was immediately rejected. She died about 9 o'clock the same morning. During the afternoon of the same day the woman who kept the house was attacked suddenly with vomiting, purging, and cramps in her legs. She was immediately subjected to efficient treatment and she soon recovered. During the following night the father of the little girl who died, was attacked also with great violence. And though I was summoned to him early, yet the disease was not arrested until he was brought to the verge of collapse. He, however, slowly recovered.

The three last cases mentioned had been residents of the city one year or more. On the 18th of May, the same day the last of the cases mentioned occurred, an Irishman who had been indulging rather freely in the use of intoxicating drinks for several days, left his house early in the morning to commence his work on the south branch of the river about one mile distant. He was attacked with violent vomiting and purging before he reached his work and immediately returned. All the phenomena of malignant cholera were rapidly developed, and when I saw him, at 10 o'clock A. M., he was pulseless and in a state of perfect collapse. He died about 6 o'clock P. M. of the same day.

From this time to the 1st of June, I saw nine cases, all but one of which was in a settlement of shanties near the east side of the North branch of the River, and between Kinzie Street and the main trunk of the River. Of these three were German emigrants, all in one filthy crowded house. Two were fatal and one recovered. The other six were Irish, inhabiting the dampest and filthiest class of shanties. Of these, however, only two died. During the same period of time several other cases occurred in the same neighborhood, which I did not see, some of which proved fatal.

On the first day of June I was called to one fatal case in the person of a most estimable lady, surrounded by all the comforts of life. She had suffered from impaired health several years, and was subject to occasional turns of diarrhœa. On the morning of April 30th she was attacked with a very moderate watery diarrhœa which continued through the day, but so moderate that she continued up and attending to her household duties. About 10 o'clock P. M., the diarrhœa continuing, she took a mixture of Syrup of Rheubarb and Paragoric, which she had in the house, and went to bed. About 11 o'clock, P. M., she vomited slightly, and from that time all the symptoms of cholera became rapidly developed. I was called about 2 o'clock, A. M., but treatment was of no avail, and she died about 5 o'clock, P. M., of the 1st of June. From this time to the 9th of June I saw only a few cases of watery diarrhœa. But from the 9th to the 13th, the date of this report, the disease has rapidly increased—twelve cases having come under my observation.

These cases have been scattered in every division of the city, but were nearly all Irish laborers—six of them have proved fatal—at least four of the number being in collapse before any medical aid was called. It will be seen by the above that the whole number of cases which have come under my observation during the past two months, is thirty-three, of which fifteen were fatal Three of them I saw only in consultation, and six others were either dying or in a state of complete collapse before medical aid was sought. I have no reliable means of ascertaing the whole number of cases which have occurred in the city during the present season. Those which have come under my observation have shown no features differing essentially from the same disease as it has appeared in former years; neither have I been able to discover any difference in the action of medicines given to control it. In regard to the general sanitary condition of the city, it is sufficient to say that the streets and alleys have been much neglected during all the spring—they are and have been, consequently, very filthy and wet. The house on Kinzie street where four cases occurred about the middle of May was dirty, crowded, especially in the lodging rooms; and the back yard and alley were literally nauseous with filth. The neighborhood near the north branch of the river, where most of the cases occurred during the last week in May was in no better condition. It will be observed, by reference to the dates I have given, that the cholera has been in the city since the middle of April. But most of the cases have occurred in groups extending over a few days, and then ceasing to occur or nearly so. By referring to my notes of the weather or atmospheric changes, I find that the six first cases detailed, occurred during four or five days of very warm, sultry weather, commencing about the 20th of April. The first half of the month had been cold, but it was followed by a few warm days, with showers and an atmosphere almost saturated with moisture. Before the close of the month it again became cold for the season and remained so until near the middle of May. During the last half of May the atmosphere was moderately warm, with occasional showers, and a high degree of saturation. On the 6th of June it changed to unusual cold with excessive fall of rain and continued so until the 9th. From the 9th to the present date it has been warm and clear, but with a high degree of moisture in the atmosphere. The connection of these atmospheric conditions with the prevalence of the disease may be readily seen by the reader. It has been impossible to trace the cases which I have seen to any connection with each other, or with the disease in other parts of the country. On the occurrence of the first fatal case mentioned in this report, I communicated it to the Mayor and Board of

Health, with the warning that indications were plainly visible, of an approaching cholera season, and with an urgent request that no time should be lost in cleansing and draining the streets and alleys; and in scattering through the filthiest portions a few tons of lime. But up to the present time *nothing* has been done; and the prospect is that your committee will have abundant materials for a much more extended report at our next monthly meeting.

Chicago, June 13th, 1854.

